# *Coprinopsis cinerea* Enhances Corn Straw Degradation Efficiency in Association with Remodeled Bacterial Communities and Metabolic Pathways

**DOI:** 10.3390/microorganisms14102159

**Published:** 2026-09-24

**Authors:** Furan Zhang, Bing Wang, Changchun Li, Su Han, Shuai Wang, Jian Luan

**Affiliations:** Jilin Provincial Key Laboratory of Plant Resource Science and Green Production, Jilin Normal University, Siping 136000, China

**Keywords:** *Coprinopsis cinerea* (*C. cinerea*), straw degradation, bacterial community, metabolomics

## Abstract

*Coprinopsis cinerea* (*C. cinerea*) is a well-recognized model fungus for lignocellulose degradation, yet its microecological mechanism in regulating native microbiota during straw decomposition remains poorly understood. We constructed a physically separated two-layer solid-state microcosm with an upper sterilized corn straw layer and a lower unsterilized field soil layer retaining native bacterial communities, and collected samples on days 15, 30 and 45 post-inoculation for 16S rRNA sequencing, untargeted metabolomics and co-occurrence network analysis. Inoculation did not increase overall bacterial richness and temporarily reduced ASV richness at day 30, but induced marked community structure shifts: enrichment of lignocellulolytic taxa *Pseudomonas* and *Flavobacterium*, reduction in Acidobacteriota, and a denser, more synergistic microbial network at the mid-stage. Metabolically, inoculation enhanced early phenylalanine metabolism linked to lignin degradation, and mid- to late-stage ABC transporter, glycolysis/gluconeogenesis and purine metabolism pathways; by day 45, cellulose and hemicellulose degradation rates were 13% and 29% higher in the inoculated group. In conclusion, *C. cinerea* enhances straw lignocellulose degradation via multi-level microecological remodeling of bacterial communities and metabolic pathways as well as potential direct enzymatic contributions, providing a reference for developing microbiome-modulating fungal inoculants. This study is limited to bacterial responses, with fungal community dynamics uncharacterized.

## 1. Introduction

Corn straw is one of the most abundant lignocellulosic substrates in terrestrial ecosystems, and microbial degradation of lignocellulosic biomass is a long-standing core issue in environmental microbiology [1]. The recalcitrance of lignocellulose structure limits the efficiency of natural microbial decomposition, which has become a bottleneck restricting the bioconversion of straw biomass worldwide. In China, Northeast China (centered in Jilin Province) is a major maize-producing region that generates approximately 90 million tons of corn straw annually, with Jilin Province alone accounting for over 35 million tons per year; microbial-mediated straw decomposition is an important approach for in-situ straw valorization. Chemically, corn straw has a rigid cross-linked structure composed of cellulose, hemicellulose and lignin, which confers strong natural recalcitrance to biodegradation and leads to slow natural decomposition [2]. Native microorganisms in farmland soils alone are insufficient to achieve complete straw degradation, which greatly limits the efficiency of straw return and feed utilization. Conventional physical and chemical pretreatments are generally limited by high energy consumption and secondary pollution risks [3]; by contrast, microbial degradation has emerged as a mainstream technology for straw valorization due to its environmental friendliness, low cost and feasibility for in-situ field application [4].

Current development of straw-degrading microbial inoculants is largely focused on screening single degrading strains and optimizing extracellular enzyme profiles [5]. Most existing studies emphasize the degradation performance of purely cultured strains, but overlook the interaction dynamics of microbial communities in complex agro-ecosystems. This gap has led to common drawbacks of commercial inoculants: unstable field colonization, vulnerability to antagonism by native microorganisms, and insufficient long-term degradation efficacy, which hinder large-scale field application [6]. Therefore, moving beyond the single-strain research paradigm, it is critical to identify core fungal strains that combine both lignocellulose-degrading activity and community-modulating functions, and to decipher the microecological mechanisms by which they may reshape native microbial communities, regulate metabolic processes, and accelerate straw decomposition. This remains a key research priority in the field of straw biovalorization [7].

*Coprinopsis cinerea* (*C. cinerea*) is a well-recognized model saprophytic basidiomycete, with a clear genetic background, mature genetic manipulation tools, and established liquid/solid culture systems. A full set of DNA transformation and CRISPR gene editing platforms have been developed for this fungus, which facilitate the dissection of lignocellulose metabolic regulatory pathways [8,9,10]. Its genome encodes a complete repertoire of lignocellulolytic enzymes, including laccases, cellulases and hemicellulases. With rapid mycelial growth and broad environmental adaptability, *C. cinerea* is suitable for large-scale inoculum fermentation and in-situ application to field straw [11,12] making it an ideal model for investigating cross-kingdom fungal-bacterial synergistic degradation of straw [7,13]. To date, most studies on *C. cinerea* have been restricted to enzyme activity assays in pure culture and static degradation evaluation on single straw substrates [14,15,16]. Only a few co-culture studies have demonstrated that this fungus can communicate with bacteria via signaling molecules, altering bacterial growth and community composition [17]. However, systematic multi-omics analyses are still lacking regarding how *C. cinerea* dynamically drives the succession of native bacterial communities and temporally reprograms microbial metabolic pathways after colonizing straw substrates. The complete mechanistic chain from “inherent degradative traits of the strain” to “native microbial community remodeling”, “cross-kingdom metabolic synergy” and “enhanced straw degradation efficiency” has not been established. This mechanistic gap substantially limits the large-scale application of this fungus in straw feed production and straw return practices [7,18].

In this study, we used *C. cinerea* as the experimental strain to systematically investigate the physiological and microecological synergistic mechanisms underlying corn straw degradation. We first optimized culture conditions to achieve large-scale inoculum production. Then, by integrating 16S rRNA high-throughput sequencing, untargeted metabolomics and microbial co-occurrence network analysis, we elucidated the role of *C. cinerea* in reshaping the composition, interspecific interactions and metabolic functions of soil bacterial communities, and quantified the enhancement of straw degradation via fiber component analysis. This study aims to clarify whether, in a single-fungus, corn-straw, physically separated laboratory microcosm, *C. cinerea* is associated with efficient straw degradation and concurrent reshaping of native bacterial communities and metabolic pathways, providing a theoretical basis for the development of long-acting straw-decomposing microbial inoculants with microbiome-modulating functions. The contribution is therefore specific to this controlled system rather than a general mechanism of fungal-mediated lignocellulose degradation.

## 2. Materials and Methods

### 2.1. Lignocellulose-Degrading Microorganisms

The *Coprinopsis cinerea* (*C. cinerea*) strain used in this study was isolated from farmland black soil at the experimental field of Jilin Normal University (Siping, Jilin Province, China; E 124°34′, N 43°15′). This strain is maintained as a laboratory stock at the Jilin Provincial Key Laboratory of Plant Resource Science and Green Production, Jilin Normal University, and is available for academic research upon request. Preliminary functional characterization confirmed that this strain secretes a complete set of lignocellulolytic enzymes (laccase, cellulase and xylanase) and exhibits strong environmental adaptability to soil microhabitats.

### 2.2. Construction of Solid-State Microcosm System, Sample Collection and Determination of Degradation Indicators

A laboratory-scale open solid-state microcosm system was constructed to investigate microecological interactions during corn straw decomposition. The reactor was separated into two layers by a hollow elastic partition, which enabled gas exchange and microecological interaction between layers while avoiding direct substrate contact. Ventilation holes were reserved at the bottom and top of the device to maintain an open aerobic incubation environment.

The lower layer was filled with 1.5 kg of non-sterile field soil collected from local maize farmland (Siping, Jilin, China), which served as the source of intact indigenous soil microbial communities. A nylon mesh bag was placed in the upper layer, loaded with 400 g corn straw fragments (particle size < 5 mm) mixed with 100 g humus soil (decomposed organic matter from forest topsoil, used to adjust substrate porosity and basal nutrient status). The upper straw–humus mixture was autoclaved at 121 °C and 0.1 MPa for 30 min to eliminate intrinsic microorganisms, ensuring an initially sterile upper degradation system. The substrate moisture content was adjusted to 35 ± 5% using sterile distilled water [19].

For inoculation, the spore suspension of *C. cinerea* was prepared and activated as follows: cultured spores were centrifuged at 8000× *g* for 10 min, resuspended in sterile PBS to adjust OD_600_ to 1.0, and pre-cultured in PSB medium at 28 °C and 180 rpm for 12 h. The pretreatment group (Cc) was inoculated with 50 mL activated spore suspension into the upper nylon bag substrate, while the control group (CK) received an equal volume of sterile PBS without fungal inoculation.

All microcosms were incubated at 25 ± 3 °C and 55 ± 10% relative humidity. Samples were collected on days 15, 30, and 45 after inoculation, generating six treatment groups (CK15, CK30, CK45, Cc15, Cc30, Cc45), each with three biological replicates. For microbial community sequencing and untargeted metabolomic analysis, approximately 10 g of soil samples were aseptically collected from the non-sterilized lower-layer soil of each reactor, to characterize dynamic responses of bulk-soil indigenous microbes to fungal-mediated straw decomposition. The basic physicochemical properties of the original field soil were as follows: pH 6.67, organic matter 12.4 g/kg, total nitrogen 89 mg/kg, total phosphorus 20.4 mg/kg, and available potassium 197 mg/kg.

For lignocellulosic component determination, the upper straw–humus substrate in nylon bags was harvested, freeze-dried using a Christ Alpha 1–4 LDplus freeze dryer (Christ, Osterode, Germany), and pulverized with a Retsch ball mill (Retsch, Haan, Germany) for subsequent structural and compositional analysis.

The contents of cellulose, hemicellulose, and lignin were determined via the Van Soest detergent fiber method [20]. Briefly, neutral detergent fiber (NDF), acid detergent fiber (ADF), and acid detergent lignin (ADL) were measured sequentially. Hemicellulose content was calculated as NDF − ADF, cellulose content as ADF − ADL, and lignin content as ADL. The degradation rate of each component was calculated as: (initial content − residual content)/initial content × 100%.

### 2.3. Soil DNA Extraction and 16S rRNA High-Throughput Sequencing

Bacterial community high-throughput sequencing was performed to investigate microbial succession characteristics during fungal pretreatment. Approximately 5 g of unsterilized lower-layer soil was collected from each replicate sample, immediately frozen in liquid nitrogen, and transported on dry ice to Beijing Baimike Biotechnology Co., Ltd. (Beijing, China) for sequencing. Total genomic DNA was extracted using the TGuide S96 Magnetic Bead Soil/Fecal Genomic DNA Extraction Kit (Beijing Baimike Biotechnology Co., Ltd., Beijing, China). The V3–V4 variable regions of bacterial 16S rRNA genes were amplified using specific primers (Table 1).

The PCR amplification procedure was set as follows: pre-denaturation at 95 °C for 5 min; 25 cycles of denaturation at 95 °C for 30 s, annealing at 50 °C for 30 s, and extension at 72 °C for 40 s; final extension at 72 °C for 7 min, and preservation at 4 °C. Amplicon integrity was verified via 1.8% agarose gel electrophoresis. Qualified libraries were sequenced on the Illumina NovaSeq 6000 platform (Illumina Inc., San Diego, CA, USA) with 10% PhiX as the internal control [21].

Raw sequencing data were quality-filtered using Trimmomatic v0.33, and primer sequences and poly-G tails were removed using Cutadapt v1.9.1. Sequence denoising, pairing, and chimera removal were performed via the DADA2 plugin in QIIME2 v2020.6 to obtain amplicon sequence variants (ASVs) [22,23]. Taxonomic annotation of ASVs was conducted based on the SILVA database. Prokaryotic phylum names in this study comply with the official nomenclature standard proposed by Oren and Garrity (2021, https://doi.org/10.1099/ijsem.0.005056). Alpha diversity indices including Chao1, Shannon and Simpson were calculated to analyze bacterial community dynamics during straw pretreatment.

### 2.4. Determination of Soil Metabolites

Untargeted metabolomic analysis was conducted to explore the molecular regulatory characteristics of indigenous soil microorganisms responding to fungal pretreatment. Approximately 100 mg of freeze-dried lower-layer soil sample was transferred to a 2 mL centrifuge tube, mixed with pre-cooled methanol and steel beads, and vortexed for 35 s. The mixture was ground by a tissue grinder at 55 Hz for 60 s and ultrasonicated for 10 min, then incubated at −20 °C for 20 min. After centrifugation at 12,000 rpm and 4 °C for 8 min, the supernatant was filtered through a 0.22 μm membrane for LC-MS detection. Equal volumes of all sample extracts were mixed to prepare quality control (QC) samples for instrument stability monitoring.

Metabolomic detection was performed on an Ultimate 3000 ultra-high-performance liquid chromatography system (Thermo Fisher Scientific, Waltham, MA, USA) coupled with an Orbitrap Exploris 480 high-resolution mass spectrometer (Thermo Fisher Scientific, Waltham, MA, USA), equipped with an ACQUITY UPLC^®^ HSS T3 column (Waters Corp., Milford, MA, USA) (2.1 × 100 mm, 1.8 μm). The column temperature was 40 °C, the sample tray temperature was 4 °C, and the injection volume was 2 μL. Electrospray ionization was operated in both positive and negative modes with spray voltages of 3.8 kV and −3.4 kV, respectively. The capillary temperature was 320 °C, the auxiliary gas temperature was 300 °C, the sheath gas flow rate was 40 arb, and the auxiliary gas flow rate was 15 arb. The MS1 resolution was 60,000 and the MS2 resolution was 15,000, with stepped collision energies of 20/30/40 eV.

Raw mass spectrometry data were processed using CD search software for peak alignment and metabolite extraction. Metabolite annotation was performed based on mzCloud, MZvault, and Chemspider databases. KEGG pathway enrichment analysis of differential metabolites was implemented using clusterProfiler v3.0.5 [24].

### 2.5. Statistical Analysis

All statistical analyses were performed using SPSS 26.0 (IBM, Armonk, NY, USA). Independent sample *t*-tests were used to compare differences between the CK and Cc groups at the same sampling time point (marked with asterisks in the figures), and the non-parametric Mann–Whitney U test was adopted when data failed the normality test or homogeneity of variance test; one-way ANOVA coupled with Fisher’s least significant difference (Fisher-LSD) post-hoc multiple comparison method was applied to analyze differences in indicators across different sampling time points within the same treatment group (marked with lowercase letters in the figures). A *p*-value < 0.05 was considered statistically significant, and all experimental results are presented as mean ± standard deviation (mean ± SD).

## 3. Results

### 3.1. Dynamics of Straw Component Degradation

The initial substrate contained 35.6% cellulose, 22.3% hemicellulose, and 21.9% lignin. Over the 45-day incubation period, cellulose and hemicellulose contents in both groups showed an overall downward trend over the 45-day incubation period (Figure 1A,B). From day 15 onward, the Cc group exhibited significantly lower cellulose and hemicellulose residues than the CK group at each sampling time point (*p* < 0.05). Two-way ANOVA revealed that both inoculation treatment and incubation time exerted significant effects on cellulose and hemicellulose degradation efficiencies (all *p* < 0.01), with a significant interaction effect on hemicellulose degradation efficiency (*p* < 0.05). By the end of incubation, the degradation efficiencies of cellulose and hemicellulose in the Cc group were 13% and 29% higher than those in the CK group, respectively.

The relative lignin content increased gradually in both treatments (Figure 1C). This pattern arose from the preferential and rapid degradation of cellulose and hemicellulose, which reduced the total dry weight of the substrate and caused relative enrichment of lignin in the residues. Significantly higher relative lignin content was observed in the Cc group on days 30 and 45 (*p* < 0.05). This difference is driven by two combined effects: first, *C. cinerea* inoculation accelerated the selective degradation of cellulose and hemicellulose, so that residual lignin was further concentrated as total substrate mass declined faster than in the control; second, aromatic components from accumulated fungal mycelium and repolymerized lignin intermediates are co-detected in the standard gravimetric lignin assay, which also increases the measured relative lignin content in the inoculated group.

The dynamic degradation efficiencies of cellulose and hemicellulose are presented in Figure 1. The Cc group consistently outperformed the CK group in cellulose degradation efficiency across the entire incubation period (Figure 1D). Hemicellulose degradation responded more rapidly: within the first 15 days, hemicellulose degradation efficiency in the Cc group increased sharply and was significantly higher than that in the CK group (Figure 1E). These functional results served as phenotypic evidence for the subsequent microecological mechanism analysis.

### 3.2. Assessment of Bacterial Community Richness

To reveal the microecological mechanism underlying the enhanced degradative function, we first assessed the effect of *C. cinerea* inoculation on bacterial community richness by comparing the number of observed ASVs across different treatments and time points (Figure 2). At the early stage (15 d), there was no significant difference in observed ASV numbers between the Cc treatment group and the CK control group. As the degradation process progressed, by day 30, the observed ASV number in the Cc treatment group was significantly lower than that in the CK control group. By day 45, no significant difference in observed ASV numbers was detected between the two groups. This transient reduction in richness was consistent with directional filtering of the bacterial community associated with fungal inoculation, rather than a persistent loss of taxonomic diversity.

### 3.3. Shifts in Bacterial Community Alpha Diversity

To investigate the effects of *C. cinerea* inoculation on community structural stability and functional succession, this study analyzed the alpha diversity of bacterial communities at different stages (Figure 3). At day 15 (Figure 3A–C), there was no significant difference in the Chao1 index between the CK and Cc groups, but the Shannon index in the Cc group was significantly higher than in the CK group (*p* < 0.05). This suggested that fungal colonization may initially promote bacterial coexistence by releasing soluble metabolites and expanding available nutritional niches.

By day 30 (Figure 3D–F), the Shannon index and Simpson index of the Cc group were significantly lower than those of the CK group (*p* < 0.05), while the Chao1 index still showed no significant difference. By day 45 (Figure 3G–I), the Chao1 index, Shannon index, and Simpson index of the Cc group were all significantly lower than those of the CK group (*p* < 0.001). The progressive decline in diversity was consistent with directional community succession: taxa adapted to the lignocellulose-degrading microenvironment were enriched, while non-adaptive rare taxa gradually declined, a pattern that may lead to increased functional specialization of the community.

Two-way ANOVA results showed that inoculation treatment, incubation time, and their interaction all had significant effects on the Shannon and Simpson indices (*p* < 0.01). The inconsistent trends between estimated richness (Chao1) and observed ASVs at day 45 may be explained by the reduction in low-abundance rare taxa in the inoculated group, which affected the estimated total richness but had limited impact on the number of actually observed ASVs.

### 3.4. Taxonomic Composition Shifts in Bacterial Communities

To explore how *C. cinerea* inoculation reshapes bacterial community assembly, this study analyzed the dynamic changes in dominant groups at phylum, class, order, and genus levels (Figure 4).

At the phylum level (Figure 4A), Pseudomonadota was the dominant phylum throughout the incubation process in all treatments. In the CK group, the relative abundance of Pseudomonadota remained relatively stable over time; in the Cc treatment group, its abundance at days 30 and 45 was significantly higher than that of the CK group at the same time points (*p* < 0.05). Additionally, Bacteroidota in the Cc group also showed a higher trend than the CK group on day 45. Both phyla are typical copiotrophic taxa with strong carbon substrate utilization capacity. Conversely, oligotrophic phyla including Acidobacteriota and Chloroflexota, which were abundant in the CK group, were significantly suppressed in the Cc treatment group (*p* < 0.05), a pattern consistent with inoculation shifting the community toward a copiotrophic functional profile.

At the class and order levels (Figure 4B,C), the Cc treatment significantly enriched taxa with high lignocellulolytic potential, such as GammaPseudomonadota and Xanthomonadales. At the genus level (Figure 4D), the Cc treatment was associated with specific enrichment of bacterial genera including *Pseudomonas* and *Xanthomonas*, which are widely recognized for their ability to secrete carbohydrate-active enzymes and participate in synergistic degradation with fungi. The proportion of unclassified and low-abundance taxa (“Others”) was consistently lower in the Cc group than in the CK group, and decreased gradually with incubation time, confirming the directional assembly of the functional community.

### 3.5. Remodeling of Bacterial Co-Occurrence Networks

By combining network visualization (Figure 5) with quantitative topological parameters (Figure 5), this study characterized the remodeling of bacterial co-occurrence patterns. We explicitly note that these networks reflect statistical correlations in taxon abundance profiles, and do not constitute definitive proof of actual synergistic biological interactions between taxa.

At day 15, the number of network nodes (Figure 5G) in the Cc group was significantly lower than in the CK group (*p* < 0.05), consistent with the alpha diversity results. However, the number of network edges (Figure 5H) and the network average degree (Figure 5I) showed no significant differences between the two groups, a pattern consistent with remaining core species quickly establishing dense functional connections after the loss of peripheral rare taxa, and maintaining the basic connectivity of the network.

At day 30, the network average weighted degree (Figure 5J) was significantly higher in the Cc group than in the CK group (*p* < 0.05). This was consistent with interspecies synergistic associations being greatly enhanced in the inoculated group at this stage, corresponding to the tightly linked functional modules observed in the network diagram (Figure 5D). Such enhanced cooperative interactions may facilitate metabolic cross-feeding and niche complementarity among community members. Taxon-level network analysis further identified core and keystone bacterial groups in the co-occurrence patterns. Pseudomonadota was the dominant core phylum with the largest node size and the highest number of connecting edges, consistent with its high relative abundance and central functional position. At the genus level, *Pseudomonas*, *Flavobacterium* and *Sphingomonas* emerged as keystone taxa with high connectivity and betweenness centrality, which may act as hubs mediating cooperative interactions within the degradative community.

At day 45, the number of edges and average weighted degree in the Cc group declined to levels not significantly different from the CK group, while the number of nodes remained significantly lower. This suggested that the interaction network may gradually mature and stabilize at the late stage of degradation, forming a streamlined, low-redundancy interaction structure dominated by core functional taxa.

### 3.6. Temporal Shifts in Soil Metabolic Profiles

This study systematically screened differential metabolites to reveal the overall metabolic changes in the soil system after *C. cinerea* inoculation, which jointly reflect the metabolic activity of fungi, indigenous bacteria and straw decomposition processes. The volcano plot (Figure 6) showed that the number of significantly differential metabolites increased continuously with incubation time, and reached the maximum at day 45, reflecting the persistent regulatory effect of inoculation on soil microbial metabolic activity.

KEGG enrichment analysis showed distinct stage-specific functional characteristics (Figure 6). At day 15, differential metabolites were significantly enriched in pathways related to lignocellulose depolymerization, including flavonoid degradation, phenylalanine, tyrosine and tryptophan metabolism, as well as starch and sucrose metabolism and amino sugar metabolism. These pathways are consistent with the initial breakdown of straw structural components and the release of intermediate metabolites.

At day 30, ABC transporters and the phosphotransferase system became the core enriched pathways, accompanied by significant enrichment of nitrogen metabolism pathways such as arginine and proline metabolism. The upregulation of transport systems is consistent with enhanced intercellular substance exchange and nutrient uptake, which aligns with the strengthened synergistic interactions observed in the co-occurrence network.

At day 45, material transport pathways and core energy metabolism pathways including ABC transporters, purine and pyrimidine metabolism remained significantly enriched, consistent with sustained high metabolic activity of the functionally specialized community in the late incubation stage.

## 4. Discussion

### 4.1. Remodeling of Bacterial Community Assembly Enhances Lignocellulose Degradation

Alpha diversity results showed that *C. cinerea* inoculation did not increase bacterial diversity in the straw decomposition microcosm; instead, community richness declined significantly as incubation progressed. This trend is consistent with previous reports on straw-degrading microbial inoculants (Figure 2 and Figure 3) [25,26]. Exogenous saprophytic fungi have been proposed to act as an ecological filter: they may modify substrate carbon profiles, pH, and phenolic microenvironments to eliminate a large proportion of oligotrophic native bacteria lacking lignocellulolytic capacity, thereby concentrating community functions toward polysaccharide decomposition [27]. Current straw inoculant development generally relies on synthetic microbial consortia to achieve directed community domestication. However, the present data are consistent with the possibility that the single model basidiomycete *C. cinerea* may efficiently reshape the straw microhabitat and select for functional bacterial taxa, gradually establishing a stable indigenous bacterial community with specialized degradative functions. This finding is consistent with the view that single strains may exert effects beyond solely extracellular enzyme secretion [13].

Community composition analysis revealed that Cc treatment was associated with specific enrichment of Pseudomonadota (especially *Pseudomonas* within GammaPseudomonadota) and Bacteroidota (e.g., *Flavobacterium*), and with suppression of oligotrophic native phyla such as Acidobacteriota (Figure 4) [28]. As a model white-rot basidiomycete, *C. cinerea* preferentially degrades the recalcitrant lignin framework, continuously releasing phenolic small molecules and soluble reducing sugars, and may alter substrate physicochemical conditions. These changes select for degradative bacterial taxa adapted to this habitat, which may drive the directional assembly of a cross-kingdom degradative microbial community [29,30].

Co-occurrence network analysis further indicated changes consistent with optimization of community structure. At the mid-incubation stage, the inoculation group exhibited simultaneously enhanced microbial network connectivity and increased positive synergistic associations among taxa. These topological shifts were temporally aligned with the improved degradation efficiency of straw cellulose and hemicellulose (Figure 5) [31,32]. We propose that exogenous fungi may alleviate ineffective ecological competition among native microorganisms through resource partitioning, strengthen mutualistic interactions among functional taxa such as *Pseudomonas* and *Flavobacterium*, and build a metabolically complementary cooperative network. The synergistic gains from microbial interactions may further amplify straw decomposition capacity, suggesting that fungal-mediated reconstruction of microbial interaction networks may be an important microecological pathway that contributes to the enhanced degradative performance [33,34].

### 4.2. Temporal Shifts in Soil Metabolic Profiles Underpin Cross-Kingdom Interactions During Straw Decomposition

Untargeted metabolomics provided molecular-level insights into the temporal dynamics of fungal-bacterial synergistic degradation, revealing patterned shifts in metabolic pathways across different incubation stages (Figure 6) [35,36]. In the early incubation stage, differential metabolites were enriched in aromatic lignin degradation pathways such as phenylalanine metabolism, along with primary metabolic pathways including starch and amino sugar metabolism. This pattern aligns with the well-documented physiological trait of white-rot fungi to initiate lignin depolymerization prior to polysaccharide breakdown (Figure 6D) [37,38]. Phenolic signaling molecules and soluble sugars released during lignin degradation may provide essential nutrients and environmental cues for subsequent colonization by functional bacteria and directed community selection, tentatively mediating the upstream regulatory effects of fungi on the bacterial community [39].

During the middle and late incubation stages, the overall metabolic functions of the system shifted markedly. ABC transporters and core carbon and nitrogen metabolic pathways were significantly enriched, consistent with the fungal-conditioned microbial community acquiring enhanced capacities for substrate recognition, transmembrane transport, and coordinated carbon-nitrogen utilization (Figure 6E) [40]. In the late incubation stage, core energy metabolic pathways such as purine and pyrimidine metabolism remained enriched, consistent with the restructured community dominated by Pseudomonadota and Bacteroidota being able to sustain stable and high-level metabolic activity over time, consistent with the copiotrophic lifestyle of these two dominant phyla (Figure 6F) [41,42]. Time-series metabolomic evidence is consistent with a proposed continuous regulatory chain of “fungal microenvironment modification → microbial community structure reshaping → global metabolic pathway reprogramming”, providing a testable molecular ecological framework for cross-kingdom interactions during lignocellulose decomposition [35].

Collectively, the combined community and metabolomic evidence is consistent with a proposed model in which *C. cinerea* may promote efficient cellulose breakdown in corn straw through a multi-level microecological synergistic pathway: microhabitat remodeling driven by fungal metabolism → directional reshaping of the indigenous bacterial community via ecological filtering → optimization of microbial interspecific cooperative interactions → reprogramming of system-level metabolic pathways [29]. We emphasize that the causal ordering of these layers has not been directly established; the present data document associations that warrant targeted mechanistic testing. These findings suggest a microecological route by which a single model saprophytic fungus may modulate native microbiota in association with straw lignocellulose degradation, and provide a theoretical basis for the development of long-acting straw-decomposing inoculants with microbiome-regulating functions [43].

### 4.3. Limitations and Future Perspectives

Nevertheless, several limitations of this study should be acknowledged. First, as an inherent methodological feature of the double-layered incubation system, the separate contributions of direct degradation by fungal extracellular enzymes and indirect effects mediated by bacterial community remodeling cannot be completely decoupled in the current setup. Consequently, the relative contribution of each pathway, and whether the two act jointly, remains unresolved. Disentangling their respective quantitative contributions would require a more targeted sterilized soil control design, which falls outside the scope of the present study. Similarly, the physical porous barrier prevents indigenous bacteria in the lower soil layer from directly contacting the upper straw substrate. Their regulatory effect on straw degradation is mainly exerted through the bidirectional diffusion of soluble and volatile metabolic intermediates, and the specific magnitude of this indirect cross-kingdom interaction also warrants further quantitative exploration in follow-up studies. Second, all experiments were performed in simulated microcosms under controlled laboratory conditions. Accordingly, the conclusions drawn from this controlled system cannot be directly extrapolated to real farm straw-return scenarios. Natural field straw return environments are considerably more complex, with fluctuating temperature, moisture regimes and resident biotic communities that may substantially alter the colonization efficiency and degradative capacity of the inoculated *C. cinerea*. Third, this work focused exclusively on bacterial community responses, which constitutes a clear biological scope limitation of the study. The successional trajectories and functional roles of fungal communities, as well as other soil biotic components in this system, remain to be systematically elucidated. Additionally, there is a methodological limitation regarding the sterilization procedure: a single autoclaving treatment cannot achieve complete sterilization of the upper straw-humus substrate, and spore-forming bacteria may remain viable. This treatment was designed to reduce background microbial abundance rather than eliminate all microorganisms entirely, to better isolate the effects of *C. cinerea* inoculation. As both the inoculation and control groups underwent identical autoclaving procedures, this limitation does not compromise the validity of the comparative results between groups.

Building on these research boundaries, future work can be expanded in the following directions. First, a sterilized soil control system coupled with quantitative extracellular enzyme activity assays could be constructed to partition the relative contributions of direct fungal degradation and bacteria-mediated indirect effects, thereby delineating the full mechanistic chain of cross-kingdom regulation during straw decomposition. Second, field in-situ inoculation trials would be valuable to validate the practical performance of *C. cinerea* inoculants under real agricultural settings and to optimize application protocols for straw return. Third, integrated multi-omics approaches (e.g., fungal community profiling, enzyme activity quantification and non-targeted metabolomics) can be employed to more comprehensively unravel the cross-kingdom interaction mechanisms underlying straw degradation.

## 5. Conclusions

This study demonstrates that *C. cinerea* inoculation is associated with significantly enhanced corn straw lignocellulose degradation, accompanied by remodeling of native bacterial communities and temporal shifts in microbial metabolic pathways in a laboratory two-layer microcosm system. The enhanced degradation correlates with enrichment of core lignocellulolytic bacterial taxa, strengthened synergistic co-occurrence networks, and stage-specific metabolic reprogramming from lignin degradation pathways to nutrient transport and energy metabolism. These findings are limited to controlled laboratory conditions and bacterial community analyses, and field validation as well as fungal community characterization are required in follow-up work. Overall, this work provides a microecological reference for developing straw-degrading microbial inoculants with microbiome-modulating functions.

## Figures and Tables

**Figure 1:**
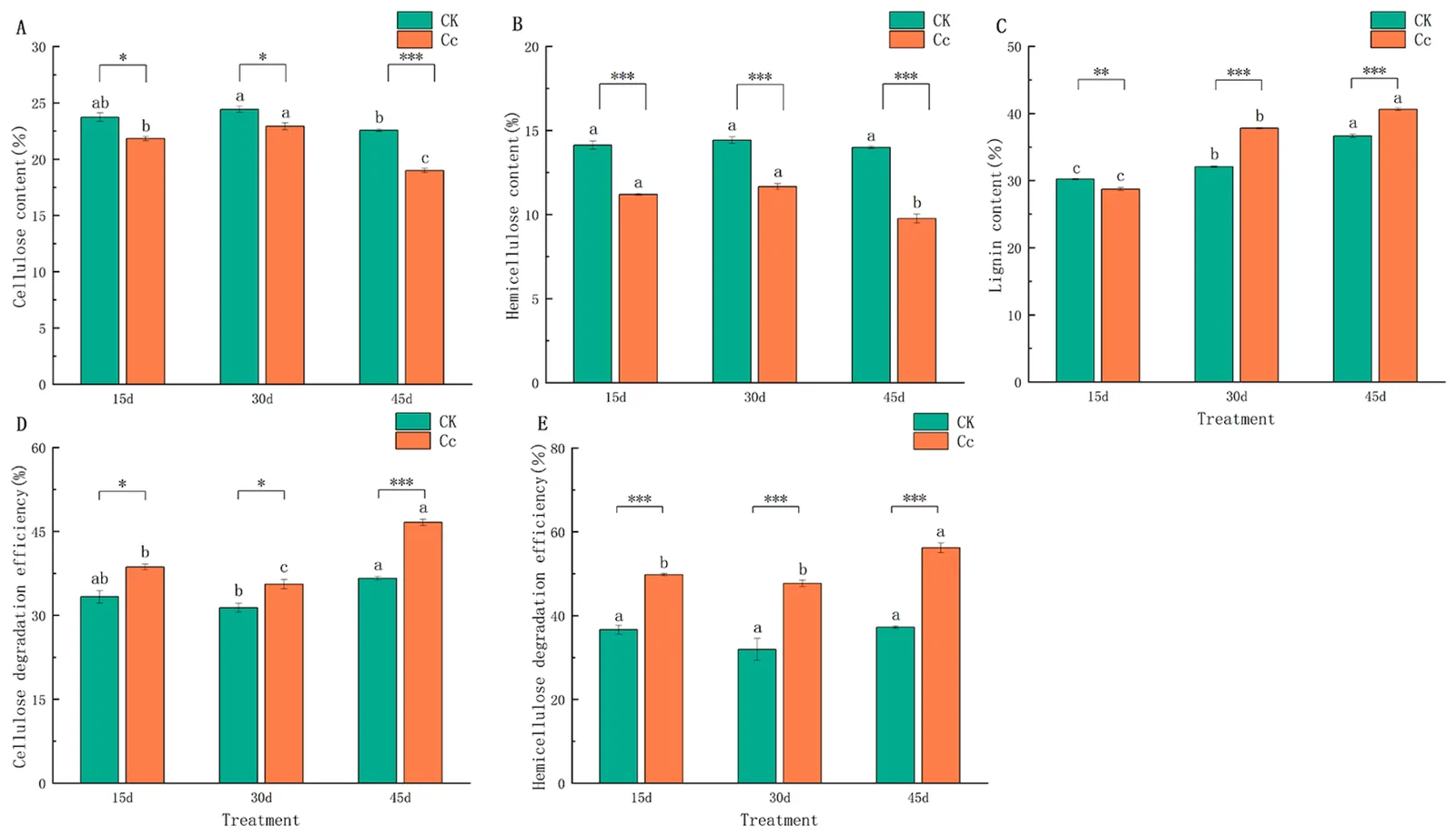
Dynamic changes in lignocellulosic component contents and degradation efficiencies in upper-layer corn straw substrate. (**A**) Cellulose content; (**B**) Hemicellulose content; (**C**) Lignin content; (**D**) Cellulose degradation efficiency; (**E**) Hemicellulose degradation efficiency. The X-axis represents pretreatment duration (15 d, 30 d, 45 d). For panels (**A**–**C**), the Y-axis indicates the mass percentage of each component relative to the dry weight of corn straw. For panels (**D**,**E**), degradation efficiency was calculated based on the absolute dry mass of each component, with correction for the total dry weight loss of the substrate. Different lowercase letters indicate significant differences among different sampling time points within the same treatment group (CK or Cc, respectively), as determined by one-way ANOVA followed by post-hoc multiple comparison test (*p* < 0.05), and groups sharing the same letter are not significantly different (*p* ≥ 0.05); asterisks indicate significant differences between the CK group and Cc group at the same sampling time point, as analyzed by independent-samples *t*-test, where * *p* < 0.05, ** *p* < 0.01, *** *p* < 0.001, and groups without asterisk notation indicate no statistically significant difference between treatments (*p* ≥ 0.05). All data are presented as mean ± standard deviation (SD), with *n* = 3 biological replicates per treatment.

**Figure 2:**
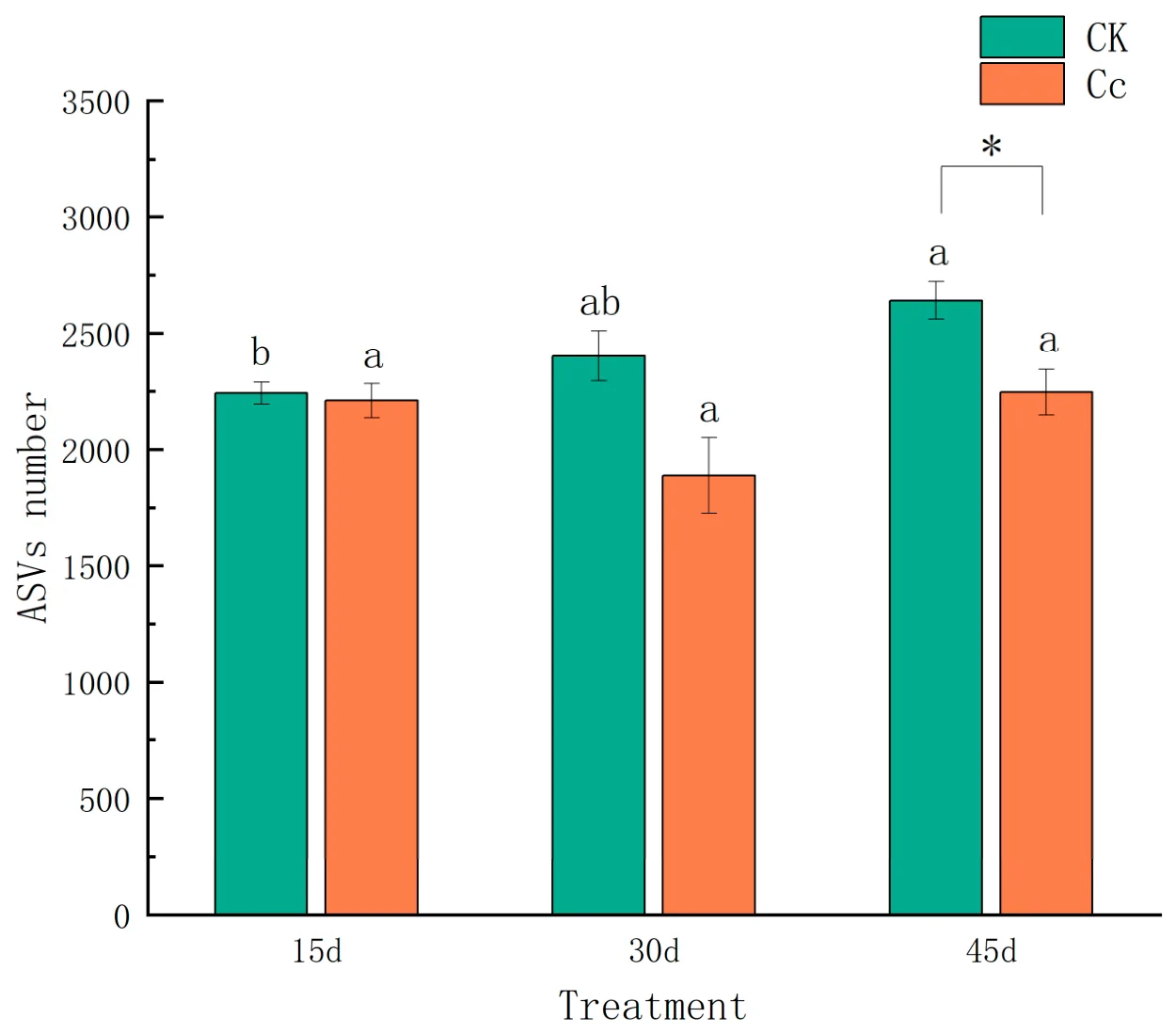
Variation in bacterial ASV richness in unsterilized lower-layer soil. X-axis: 15 d, 30 d, 45 d; Y-axis: total ASV counts. CK = uninoculated control, Cc = (*C. cinerea*). Different lowercase letters indicate significant differences among different sampling time points within the same treatment group (CK or Cc, respectively), as determined by one-way ANOVA followed by Fisher-LSD post-hoc multiple comparison test (*p* < 0.05), and groups sharing the same letter are not significantly different (*p* ≥ 0.05); asterisks indicate significant differences between the CK and Cc groups at the same sampling time point, as analyzed by independent-samples *t*-test (* *p* < 0.05), and groups without asterisk notation indicate no statistically significant difference between treatments (*p* ≥ 0.05). All data are presented as mean ± standard deviation (SD), with *n* = 3 biological replicates per treatment.

**Figure 3:**
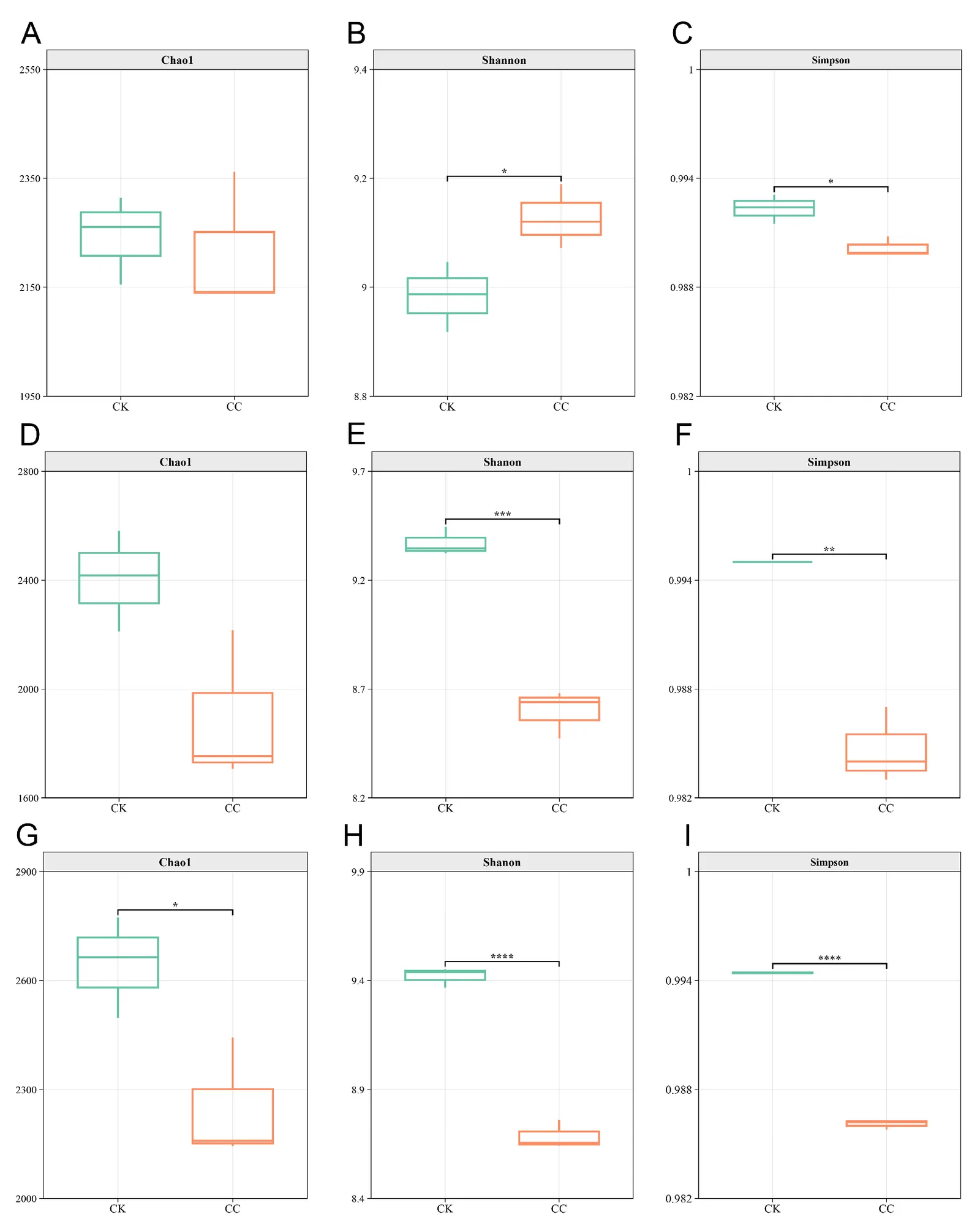
Temporal dynamics of bacterial α-diversity indices in lower reactor soil. (**A**–**C**) 15 d; (**D**–**F**) 30 d; (**G**–**I**) 45 d. (**A**,**D**,**G**) Chao1; (**B**,**E**,**H**) Shannon; (**C**,**F**,**I**). CK denotes the uninoculated control group, and CC denotes the group inoculated with *Coprinopsis cinerea* (Cc). Horizontal significance bars indicate pairwise comparisons between the CK and CC groups at the same sampling time point, as analyzed by independent-samples *t*-test (* *p* < 0.05, ** *p* < 0.01, *** *p* < 0.001, **** *p* < 0.0001); groups without asterisk notation indicate no statistically significant difference between treatments (*p* ≥ 0.05). All data are presented as mean ± standard deviation (SD), with *n* = 3 biological replicates per treatment.

**Figure 4:**
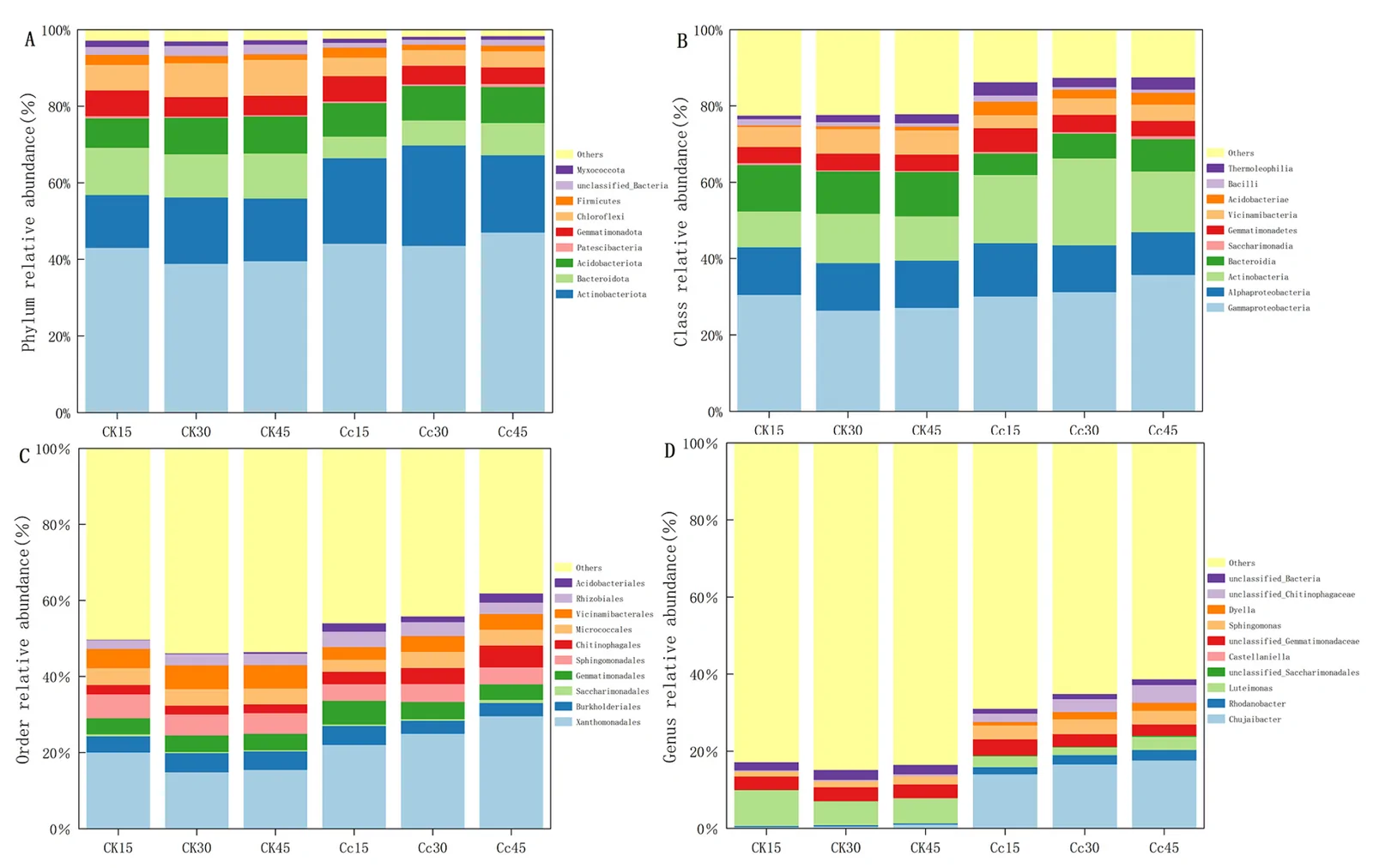
Taxonomic composition of indigenous bacterial communities in lower-layer soil. (**A**) Phylum; (**B**) Class; (**C**) Order; (**D**) Genus. X-axis: treatments and sampling time (15 d, 30 d, 45 d); Y-axis: relative abundance (%). Only top 10 abundant taxa are displayed, low-abundance taxa classified as “Others”, *n* = 3.

**Figure 5:**
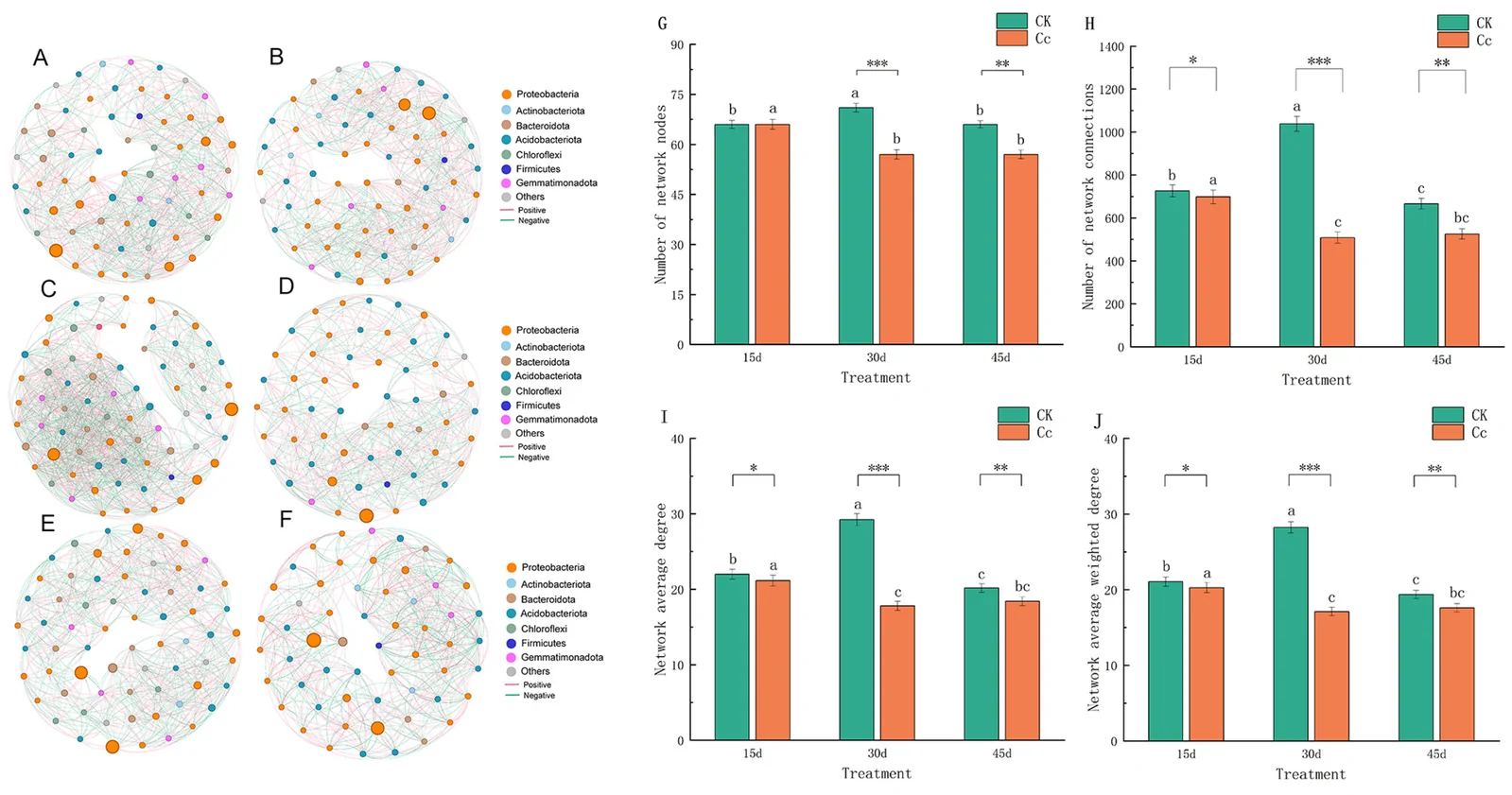
Bacterial co-occurrence networks and their topological properties in lower-layer soil. Panels (**A**–**F**) illustrate the co-occurrence networks of indigenous bacterial communities in lower-layer soil at 15 d ((**A**) CK group, (**B**) Cc group), 30 d ((**C**) CK group, (**D**) Cc group) and 45 d ((**E**) CK group, (**F**) Cc group). Red edges indicate positive correlations between bacterial taxa, while green edges indicate negative correlations; node colors correspond to distinct bacterial phyla as shown in the legend. Actinobacteriota is listed in the unified legend for consistency across all subfigures. It is absent from subplots C and D because its relative abundance was too low to meet the inclusion threshold of the co-occurrence network. Panels (**G**–**J**) present the topological characteristic parameters of the co-occurrence networks, including the number of network nodes (**G**), number of network edges (**H**), average degree (**I**) and weighted average degree (**J**). Green bars represent the CK group, and orange bars represent the Cc inoculation group. Different lowercase letters indicate significant differences across sampling time points within the same treatment group (CK or Cc, respectively), as determined by one-way ANOVA followed by Fisher-LSD post-hoc multiple comparison test (*p* < 0.05); groups sharing the same letter are not significantly different (*p* ≥ 0.05). Asterisks indicate significant differences between the CK and Cc groups at the same sampling time, as analyzed by independent-samples *t*-test (* *p* < 0.05, ** *p* < 0.01, *** *p* < 0.001); groups without asterisk notation show no statistically significant difference between treatments (*p* ≥ 0.05). All data are expressed as mean ± standard deviation (SD), with *n* = 3 biological replicates per treatment. Detailed full co-occurrence network plots for each time point are available in Supplementary Figures S1–S3 as supporting data.

**Figure 6:**
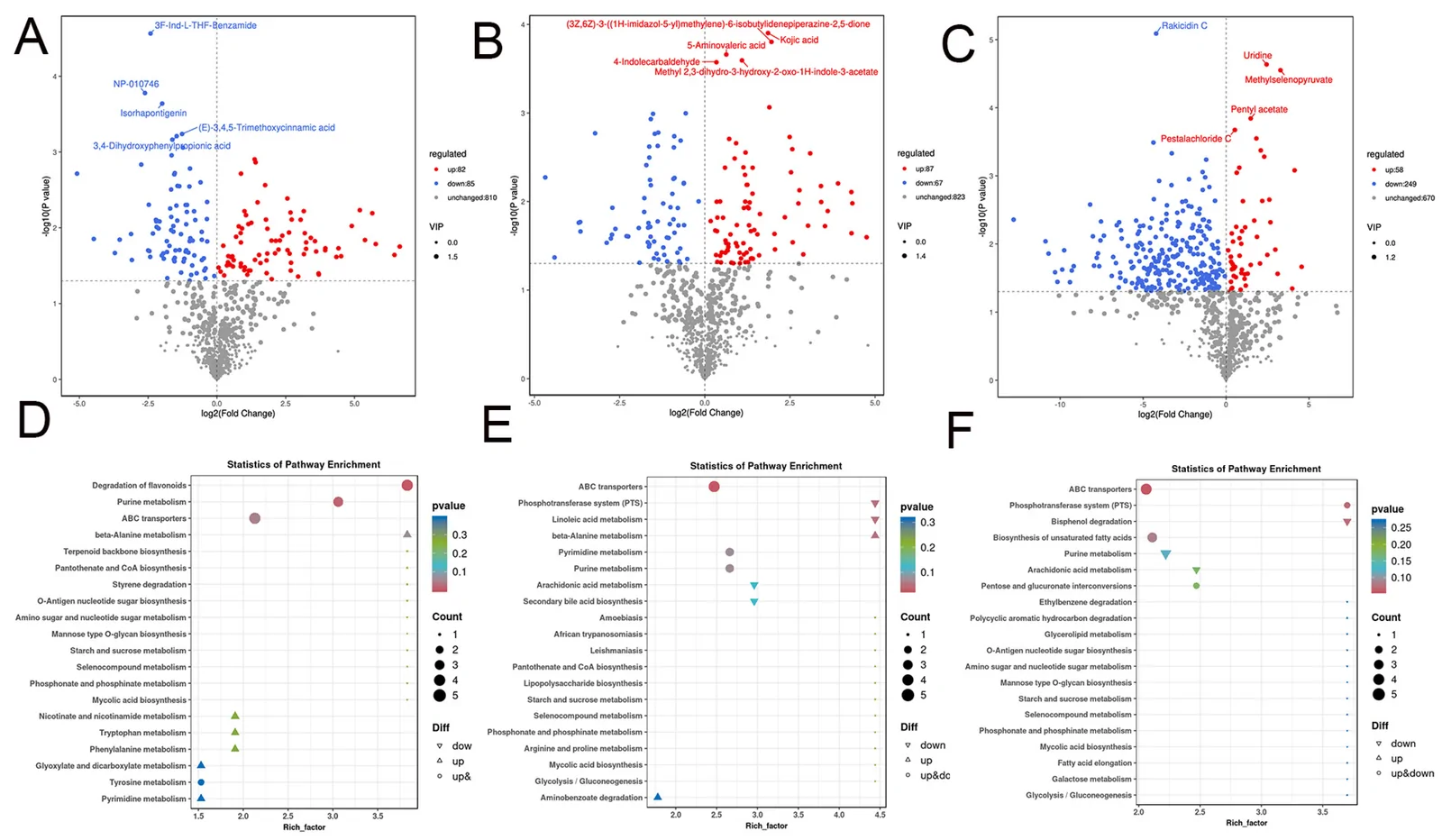
Differential metabolite profiles and KEGG pathway enrichment analysis in lower-layer soil. Panels (**A**–**C**) are volcano plots illustrating differential metabolites in lower-layer soil at 15 d (**A**), 30 d (**B**) and 45 d (**C**), in which the X-axis shows log_2_(fold change) of metabolites in the Cc group compared with the CK group, the Y-axis shows −log_10_(*p*-value), red dots represent upregulated metabolites, blue dots represent downregulated metabolites, and gray dots represent metabolites without statistically significant difference; panels (**D**–**F**) display the KEGG pathway enrichment results of these differential metabolites at 15 d (**D**), 30 d (**E**) and 45 d (**F**), where the X-axis indicates the enrichment factor of each pathway, bubble size is proportional to the count of differential metabolites enriched in the corresponding pathway, the color gradient from red to blue corresponds to decreasing statistical significance, upward triangles stand for pathways exclusively enriched with upregulated metabolites, downward triangles stand for pathways exclusively enriched with downregulated metabolites, and circles stand for pathways with bidirectional changes containing both upregulated and downregulated metabolites.

**Table 1:** Primers for bacterial 16S rRNA gene amplification.

Primer Name	Primer Sequence
16S rRNA-F	5′-ACTCCTACGGGAGGCAGCA-3′
16S rRNA-R	5′-GGACTACHVGGGTWTCTAAT-3′

## Data Availability

All data included in this study are available from the corresponding public databases cited in the article.

## Supplementary Materials

The following supporting information can be downloaded at https://www.mdpi.com/article/10.3390/microorganisms14102159/s1, Figure S1: Co-occurrence network of indigenous bacteria in lower soil at 15 d. (A) CK group; (B) Cc group. Red lines = positive correlation, green lines = negative correlation; node colors correspond to bacterial phyla in legend; Figure S2: Co-occurrence network of indigenous bacteria in lower soil at 30 d. (A) CK group; (B) Cc group. Red lines = positive correlation, green lines = negative correlation; node colors correspond to bacterial phyla in legend; Figure S3: Co-occurrence network of indigenous bacteria in lower soil at 30 d. (A) CK group; (B) Cc group. Red lines = positive correlation, green lines = negative correlation; node colors correspond to bacterial phyla in legend.

## Abbreviations

The following abbreviations are used in this manuscript:

*C. cinerea*	*Coprinopsis cinerea*
16S rRNA	16S ribosomal ribonucleic acid
PBS	phosphate-buffered saline
PSB	potato sucrose broth
NDF	neutral detergent fiber
ADF	acid detergent fiber
ADL	acid detergent lignin
ANOVA	analysis of variance
LSD	least significant difference
SD	standard deviation
ASV	amplicon sequence variant
QC	quality control
ESI	electrospray ionization
SNCE	stepped normalized collision energy
VIP	variable importance in projection
